# Collagen in the central nervous system: contributions to neurodegeneration and promise as a therapeutic target

**DOI:** 10.1186/s13024-024-00704-0

**Published:** 2024-01-25

**Authors:** Lauren K. Wareham, Robert O. Baratta, Brian J. Del Buono, Eric Schlumpf, David J. Calkins

**Affiliations:** 1 Department of Ophthalmology and Visual Sciences, Vanderbilt Eye Institute , Vanderbilt University Medical Center, 1161 21st Avenue S, 37232 Nashville, TN USA; 2 Stuart Therapeutics, Inc., 411 SE Osceola St, 34994 Stuart, FL USA

**Keywords:** Collagen, Neurodegeneration, Extracellular matrix, Alzheimer’s Disease, Glaucoma, Neuro-regeneration, Neuro-replacement, Neurovascular coupling, Collagen mimetic peptide

## Abstract

The extracellular matrix is a richly bioactive composition of substrates that provides biophysical stability, facilitates intercellular signaling, and both reflects and governs the physiological status of the local microenvironment. The matrix in the central nervous system (CNS) is far from simply an inert scaffold for mechanical support, instead conducting an active role in homeostasis and providing broad capacity for adaptation and remodeling in response to stress that otherwise would challenge equilibrium between neuronal, glial, and vascular elements. A major constituent is collagen, whose characteristic triple helical structure renders mechanical and biochemical stability to enable bidirectional crosstalk between matrix and resident cells. Multiple members of the collagen superfamily are critical to neuronal maturation and circuit formation, axon guidance, and synaptogenesis in the brain. In mature tissue, collagen interacts with other fibrous proteins and glycoproteins to sustain a three-dimensional medium through which complex networks of cells can communicate. While critical for matrix scaffolding, collagen in the CNS is also highly dynamic, with multiple binding sites for partnering matrix proteins, cell-surface receptors, and other ligands. These interactions are emerging as critical mediators of CNS disease and injury, particularly regarding changes in matrix stiffness, astrocyte recruitment and reactivity, and pro-inflammatory signaling in local microenvironments. Changes in the structure and/or deposition of collagen impact cellular signaling and tissue biomechanics in the brain, which in turn can alter cellular responses including antigenicity, angiogenesis, gliosis, and recruitment of immune-related cells. These factors, each involving matrix collagen, contribute to the limited capacity for regeneration of CNS tissue. Emerging therapeutics that attempt to rebuild the matrix using peptide fragments, including collagen-enriched scaffolds and mimetics, hold great potential to promote neural repair and regeneration. Recent evidence from our group and others indicates that repairing protease-degraded collagen helices with mimetic peptides helps restore CNS tissue and promote neuronal survival in a broad spectrum of degenerative conditions. Restoration likely involves bolstering matrix stiffness to reduce the potential for astrocyte reactivity and local inflammation as well as repairing inhibitory binding sites for immune-signaling ligands. Facilitating repair rather than endogenous replacement of collagen degraded by disease or injury may represent the next frontier in developing therapies based on protection, repair, and regeneration of neurons in the central nervous system.

## Background

Neurodegenerative disorders, both age-related and inherited, in concert with acute injuries to the CNS represent an increasingly debilitating burden to those who struggle with them, their families and care providers, and the health systems that must provide long-term care [1]. Most pharmacological or gene-therapeutic approaches to protect or repair neurons and their circuits in the CNS modulate the action of one or more receptor-ligand or intracellular signaling cascades implicated in either pathogenic pathways (to slow progression) or trophic support (to counter progression) [1]. However, outside of the cell, there may be an equally rich but underdeveloped opportunity for new therapeutic avenues. Extracellular space represents a substantial component of all tissues and organs, including the brain. A more accurate description might be extracellular *matter*, for the space is filled with an intricate and highly dynamic network of both structural and bioactive proteins that constitute the extracellular matrix (ECM) [2–4]. The most abundant protein in the ECM is collagen, which is produced in the CNS mostly by astrocytes, neurons, and vascular cells. Historically, collagen has been viewed primarily as an inert scaffolding protein that adds biomechanical stability, however, here we highlight the increasing evidence that collagen is a biologically active and integral component of the highly dynamic landscape of the CNS ECM.

## The multifaceted matrix

The ECM’s importance in physiology is reflected in its age. A key step in the evolutionary transition from unicellular to multicellular organisms was the emergence of genes coding for material components of the ECM necessary to provide an environment allowing cells to work as a unit [5, 6]. Its abundance also reflects the diverse physiological roles of the ECM, which provides structural integrity, mediates extracellular signaling capabilities, and facilitates specialization to all tissues [7]. Indeed, as cells differentiate to form specialized structures, the composition of the ECM scaffold similarly differentiates in kind to provide tissue-specific support [8]. The importance of ECM in multicellular organization is evident very early in development, as genes encoding highly conserved ECM proteins are expressed in stem cells as early as the 16-cell stage in the growing embryo [9, 10]. During the development of the CNS, ECM production is regulated spatially and temporally to drive neurogenesis, neural cell migration, and axon growth and guidance [11]. For example, in the visual system, ECM-cell communication is required to drive the connection of retinal-derived axons in the optic nerve to terminal zones in the brain [12].

In addition to its role as a substrate for tissue growth and support, the ECM also acts as an important biochemical reservoir of signaling molecules, thus allowing cells and tissues to adapt to environmental cues and stressors [5, 13]. Synthesis and release of ECM components and cell-ECM communication is an integral part of numerous biological processes including stem cell maintenance and differentiation [14], innervation [15], angiogenesis [16, 17], and wound healing [18]. Cells respond not only to the chemical composition of the ECM itself but also to its mechanical properties [19]. The ECM responds through biochemical and biomechanical signals to the resident cells of the tissue in a process termed ‘dynamic reciprocity’ or ‘bidirectional crosstalk’ [20, 21]. This terminology was first coined to define a model that described the bidirectional crosstalk between cells and their local environment [20]. The bidirectional relay of ECM-cell signaling occurs during tissue homeostasis and in pathological conditions [5]. Cells therefore must sense and regulate ECM mechanics during homeostasis to promote the structural integrity and healthy functioning of the ECM itself [19]. This process of mechanical sensing is governed through ECM proteins such as collagen and elastin which are built to withstand and respond to mechanical stretch and strain [19]. Thus, the ECM represents a highly conserved, evolutionarily critical driver of tissue specificity, connectivity, and adaption.

### The central nervous system and collagen

The complex interplay between ECM and resident cells is reflected and exemplified in the CNS, where ECM represents approximately 20% of total brain mass [22]. Both neurons and glial cells in the CNS integrate dynamically with ECM to maintain tissue homeostasis. Although neurons express and secrete various ECM components [23], astrocyte glial cells are integral to the production of ECM components including collagen and elastin [24, 25], and help to maintain ECM function and integrity as the CNS ages [26]. For example, with increasing age, the structure of the ECM in the CNS changes to regulate synaptic plasticity [27]. The ECM in the CNS predominantly comprises fibrous proteins (including collagens and elastin) and glycoproteins (including proteoglycans, glycoproteins, and laminins) that together form a three-dimensional medium through which complex networks of cells can communicate. Smaller homo- and hetero-polymers bind to form supramolecular assemblies with binding domains for growth factors, cytokines, and cell adhesion molecules [4]. In this way, the ECM serves as a medium capable of not only conveying but also binding and releasing ligands. For a more comprehensive review of ECM components in the CNS, including collagen subtypes and MMPs, we refer the reader to these recent articles [28–35].

Proteoglycans are highly abundant in neural tissue and form the basis of high-order ECM structures around cells. Proteoglycans in neural tissue are rich with covalently-bound glycosaminoglycans (GAGs)– long chains of charged polysaccharides (sugars). The major GAGs include heparin sulfate, chondroitin sulfate, hyaluronan, and keratin sulfate [11]. Even so, collagen is the most prevalent and integral component of the ECM. There are nearly 30 recognized types of collagens, and their most commonly understood role is to provide tissues with structural and mechanical integrity. In the human body as a whole, collagen is the most abundant protein, in particular types I and III [36]. Collagen renders biological stiffness, flexibility, and strength to tissues (including the ECM), influencing the degree to which stress deforms tissue and the maximum stress that can be applied before breakdown [19, 36]. Several members of the collagen superfamily, particularly collagens I, IX, and XVIII, are involved in development of the CNS, playing important roles in neuronal maturation, neural circuit formation, axon guidance, and synaptogenesis (Table 1) [37–39].

**Table 1:** Roles of collagen isoforms in CNS health and disease

CNS compartment	Collagen isoform(s)	Function in CNS	Changes with age or disease
*Development of CNS*	Collagen I, IV, IX, XVIII	Guiding neuronal outgrowth, maturation, circuit formation and synaptogenesis [37–39]	Expression compartmentalized in the CNS in adulthood
*Brain meninge basal lamina*	Collagen I, III, IV	Protective meningeal layers of the brain [40, 41]	Increased/decreased deposition of collagen IV [42]
*Vascular basal lamina*	Collagen IV, VII, XVIII	Lines blood vessels forming the blood-brain barrier	Increased deposition of collagen XVIII [43, 44], upregulation/breakdown of collagen IV [45–49]
*Perineuronal nets (PNNs)*	Collagen XIX (IV in disease states)	Stabilizing neuronal synapses	Upregulation of collagen IV production and loss of collagen XIX [50–52]

In addition to astrocyte glial cells, other cell types in the CNS in humans and mice express collagen isoforms integral to the ECM including endothelial and vascular smooth muscle cells, meningeal cells, and oligodendrocytes [40, 53]. It is worth noting that in this study, the relative levels of collagen isoform expression in oligodendrocytes, and their precursor cells is comparatively lower than in astrocytes and neurons. In addition, overall expression by oligodendrocytes is much lower in human brain tissue than is observed in mice suggesting inherent differences in collagen expression between species [53].

During development, meningeal cells are crucial for the formation of the protective meninges of the brain [40]. The meningeal layers are formed of ECM material which include high levels of fibrous collagens (including collagens I, III, and IV) expressed and secreted by meningeal cells [40, 41]. Oligodendrocytes and their precursor cells (OPCs) are also important in CNS brain development and after injury [54]. OPCs proliferate to populate the brain and spinal cord where they differentiate into mature oligodendrocytes that myelinate developing axons and axons damaged due to disease [54]. Collagen (type III [55]) and collagenases such as MMPs in the ECM can impact OPC migration and differentiation, indicating that collagen composition within the ECM is important for these processes [34].

Although microglial cells are not reported to express collagen isoforms [53], they have an indirect role in regulating ECM collagen through their production of MMPs, including MMP-9 which mediates breakdown of collagen IV in the vascular basal lamina [56, 57]. Thus, microglia, although not integral to the expression of ECM components, are an important cell type within the CNS capable of transforming the regional composition of the ECM.

The ECM of the CNS is not a uniform, homogeneous sea filling in between neuronal, vascular, and glial elements. Rather, it comprises structurally distinct and specialized landscapes or regions whose structure reflects the function of the surrounding elements. These begin with the non-fibrillar but protein-rich basal lamina. Basal lamina acts as a macromolecular sieve-like barrier between tissues, shielding cells from unwanted biochemical and biophysical stressors while also providing a medium for intercellular communication and a structure upon which epithelial or endothelial cells subsist [58, 59]. The vascular basal lamina of the CNS is produced by microcapillary endothelial cells, astrocytes, and pericytes [42]. Biochemically, the basal lamina contains four major ECM proteins: collagen IV, laminin, nidogen, and heparan sulfate proteoglycans (HSPGs) [42]. Other constituents include fibulins, osteonectin, netrin-4, and sometimes collagen XVIII [60]. The basal lamina assembles near cell surfaces– composed mainly of interconnected polymers of collagen IV and VII, with areas bound to laminin and other glycoproteins [61, 62]. Collagen IV, which is produced by endothelial cells, astrocytes, and vascular pericytes, creates a main structural scaffold to which other ECM-associated proteins can bind and interact [59, 61]. The vascular basal lamina is also found in the peripheral nervous system but differs from the CNS due to the absence of astrocytes. In the PNS, Schwann cells secrete and surround themselves with a basal lamina surface [63]; this is in contrary to oligodendrocytes of the CNS which lack the intrinsic ability to produce basal lamina components [7].

Scaffolding proteins, including fibrillar collagens, were thought only to exist along blood vessels and meninges of the brain in healthy tissue. Further, it was thought that post-development, collagen secretion in the CNS by astrocytes, neuronal cells, and other glial cells was suppressed [39]. However, the presence of collagens in healthy human brain parenchyma (including in neurons) counters these presumptions [64, 65]. As well, in the aging human brain, both neurons and astrocytes express genes encoding a range of collagen types [53, 66]. Although not a major component, collagen appears to have an important role in the structure and physiology of perineuronal nets (PNNs). Densely packed PNNs, which were wrongly identified as artifacts in Cajal’s silver staining, are condensed ECM surrounding the soma and the proximal/middle dendrites of neurons and often extend to include the axon initial segment. In the brain, they exist in multiple regions including the hippocampus, cerebral cortex, cerebellum, and basal ganglia [53]. Synapses within these regions are surrounded by and embedded in the PNN [67]. Loss of collagen XIX leads to a reduction in PNN formation in the mammalian telencephalon [50]; collagen within hippocampal PNNs is associated with the formation of long-term memory [51]. After focal ischemia in the brain, upregulation of collagen IV is evident in PNNs [52].

### Dynamic collagen: more than a scaffold

**Fig. 1:**
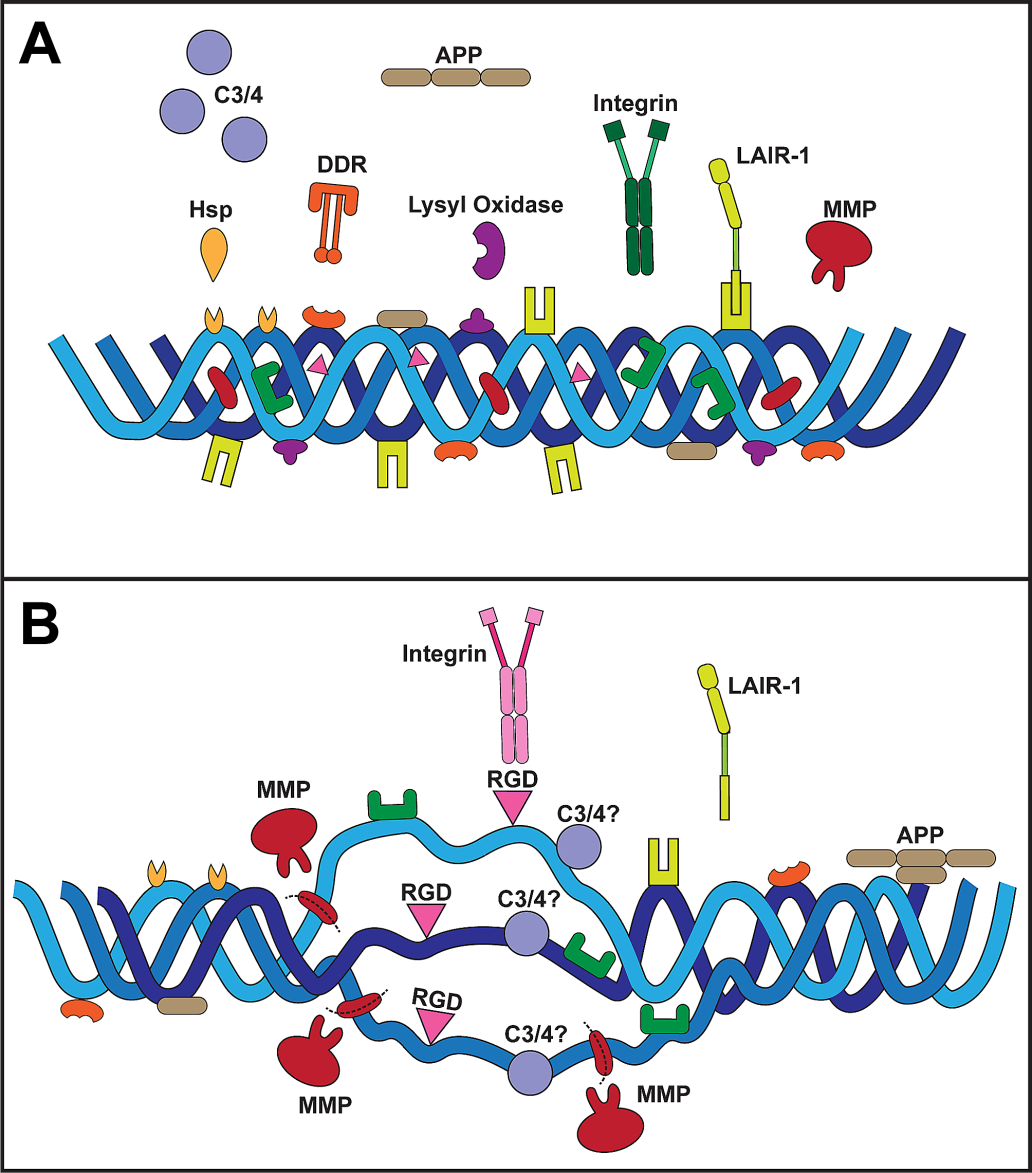
Collagen structure and ligand binding. **(A)** Collagen in its native triple helical conformation contains both exposed and hidden ligand binding sites. Binding pockets for chaperone proteins (e.g., Hsp), DDRs, and lysyl oxidase enzymes are present on the triple helical surface. Similarly, surface binding sites bind to sequester LAIR-1. APP binding sites are present on the surface of collagen I. Binding pockets for MMPs and integrins are not exposed to prevent excessive collagen breakdown or downstream signaling. **(B)** With disease or age, the activity of MMPs is elevated leading to increased collagen degradation. Breakdown of collagen exposes additional binding sites (whose exact locations are largely unknown), including RGD domains for a sub-group of integrins. LAIR-1 binding decreases in disease leading to increased immune cell activation. Based on evidence of complement C3 and C4 binding to collagen in disease, we propose the actual binding sites become available following collagen damage, though this is yet to be determined

Though fulfilling its biological role as a scaffolding protein in the ECM, collagen is nevertheless highly dynamic throughout its lifecycle, interacting with molecular binding partners by forming a complex known as the *collagen interactome* [68]. During collagen biosynthesis, interactions with enzymes such as hydroxylase and lysyl oxidases as well as chaperone proteins ensure that collagen adopts its triple helical structure as it integrates into the matrix (Fig. 1) [69, 70]. Once fully formed and integrated, collagen interacts with a number of other ECM components such as fibronectin, proteoglycans, GAGs, and heparin [69]. Collagen also binds multiple cell-surface receptors, including integrins [71], osteoblast receptors (e.g., OSCAR) [72], mannose receptors [73], and discoidin domain receptors (DDRs) (Fig. 1A) [69, 74]. During turnover, expected remodeling, or breakdown, collagen also interacts with matrix metalloproteinase (MMP) enzymes which help facilitate its degradation or digestion (Fig. 1B) [75]. Thus, MMP activity is by necessity a tightly controlled process. During disease or with increasing age, an imbalance in MMP activity (i.e., increased or decreased beyond native collagen turnover requirements) can lead to excessive matrix collagen deposition or, in the other direction, degradation [76, 77].

Collagens are multi-domain proteins having at least one triple-helical domain that can comprise most of its structure, as with collagen type I, or a much smaller fraction, as with collagen XII [78]. Many other proteins with relevance to the CNS contain collagen-like domains. The conformational state of collagen determines its binding affinity for specific ligands. Some ligands favor binding to the native triple helical collagen structure, with no affinity for fragmented collagen or smaller collagen fragments [68, 79]. For instance, some ligands including integrins, DDR1/2, MMP1, and chaperone heat shock proteins such as Hsp47 [80] bind only to the native helical structure of collagen (Fig. 1A) [68]. The collagen-binding site of DDR receptors recognizes specific surface triple helical sequences on fibrillar collagen [81] thought to be mainly obscured in the native structure of collagen to prevent over-activation of downstream signaling cascades [74, 82]. DDR1 is activated by both fibrillar and non-fibrillar collagens (types I to VI) whereas DDR2 is only activated by fibrillar collagen (types I and III) [83]. Integrin receptors lie on the outer surface of collagen [84]. However, unraveling of collagen strands post-MMP digestion exposes tri-amino RGD (arginine-glycine-aspartate) sequences in collagen that bind to specific types of integrin e.g., α_v_β_3_ (Fig. 1B) [85–87]. The molecular chaperone Hsp47 binds to only a few GXR motifs in collagen, with most of the HSP47 binding sites located near the N-terminal part of the triple-helical region [88]. Interestingly, MMPs recognize native collagen but have intrinsic helicase activity that unwinds the triple helical structure to expose MMP binding sites for the cleavage of collagen [89], an essential part of the degradation or digestion process of collagen. Other proteins, such as lysyl oxidase, preferentially bind to higher-order structures such as fibrils [90, 91]. In conditions where collagen becomes denatured or damaged, proteins involved in collagen turnover recognize epitopes exposed in the denatured or degraded collagen [68, 75]. Similarly with amyloid precursor protein (APP); binding of APP to collagen I occurs in native and damaged states and appears to be competitive with heparin– this suggests an overlap between the binding site for APP and heparin [92].

The tightly wound triple helical structure of collagen renders it weakly antigenic (the ability to *bind* to antibodies) and largely non-immunogenic (the ability to *induce* an immune response through antibodies [93]). However, the biological reactivity of collagen with cell surface antigens and receptors is becoming increasingly recognized as critical to disease states [68]. Changes to collagen structure, such as damage or increasing turnover during disease, can alter its antigenicity and immunogenicity [94, 95]. Increasing collagen turnover, as well as outright collagen damage, is evident during inflammation in disease and may represent an important therapeutic target in neurodegenerative CNS disorders [96].

### The collagenous ECM landscape as a driver of CNS disease

The ECM’s role as a biologically active microenvironment in the CNS provides biophysical stability and structure and acts as a mediator for the diffusion and availability of signaling molecules, such as those mediating interactions between axons and astrocytes [7]. Cells of the CNS modify the production and excretion of ECM components in response to environmental cues that include oxygen or nutrient concentrations and biochemical and mechanotransducive signals. In this sense, regulation of ECM turnover is critical to the function and survival of neurons in the CNS.

The extracellular landscape of the CNS evolves through changes in activity of both ECM-related genes and enzymes in both disease and aging [3]. Cellular senescence, accompanied by its reduced collagen production, is emerging as a key contributor to neurodegenerative diseases of the CNS. Cells that become senescent have a secretome that includes cytokines and chemokines, as well as ECM, that can signal to surrounding tissue [76]. Outside of the CNS in fibrotic diseases, the ECM– including collagen– can regulate cellular senescence [77]. Alterations in ECM occur across the spectrum of CNS diseases including Alzheimer’s Disease [97, 98], Parkinson’s Disease [99, 100], and of special interest to the authors, optic neuropathy [101, 102]. Of relevance to Alzheimer’s Disease, amyloid-beta (Aβ) peptides contain collagen type XXV (also known as collagen-like amyloidogenic component), which influences amyloid fibril elongation [103], and there is a genetic association between this collagen and Alzheimer’s Disease in some populations [104]. Collagen IV deposition is upregulated in microvessels of brains from Alzheimer’s patients [105]. Finally, type I collagen contains binding domains for amyloid precursor protein that contribute to monocyte recruitment in disease states [106]. Conversely, collagen VI expression in the brains of hAPP mice and individuals with Alzheimer’s Disease may be neuroprotective; increased expression of collagen VI in neurons was protective against Aβ toxicity [107]. Evidence for changes in the levels of collagen IV in basal lamina, i.e., its upregulation/depletion, in CNS diseases and with aging remain conflicting in the literature [42], nonetheless, it is the disruption of collagen IV homeostasis that appears to be fundamental to pathology.

**Fig. 2:**
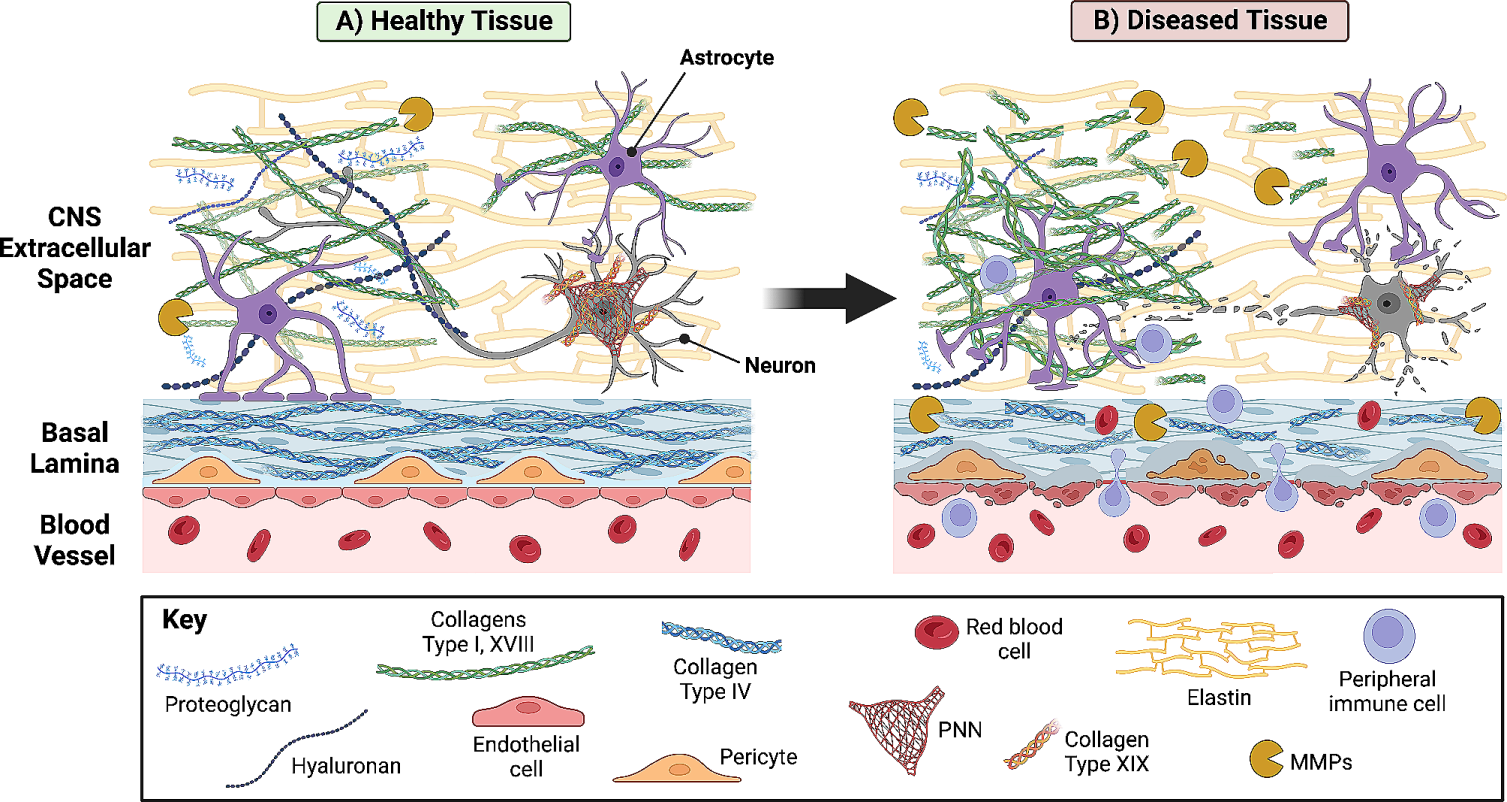
ECM collagen in healthy and diseased CNS tissue. **(A)** In healthy CNS tissue, the collagen ECM landscape is highly dynamic. In the basal lamina, collagen IV, produced by endothelial cells, astrocytes, and vascular pericytes, creates a main structural scaffold to which other ECM-associated proteins can bind and interact. In the extracellular space, collagen content varies through time. In early CNS development, collagens I, IX, and XVIII are important for differentiation and vessel development. In PNNs, collagen XIX appears to play a role in long-term memory. Matrix metalloproteinases (MMPs) are key to collagen turnover. **(B)** In diseased tissue, damaged collagen is present due to up-regulation of MMPs. Damage to collagen in the basal lamina contributes to the breakdown of the blood-brain barrier, leading to infiltration of peripheral immune cells. Increased deposition of collagen by glial cells and the breakdown of collagen by MMPs alters the biomechanical properties and ligand binding capacity of the ECM leading to increased inflammation and degeneration of neurons. Created with Biorender.com

Astrocyte glial cells in the CNS are key players in the maintenance of ECM. During disease and with aging, astrocyte physiology moves towards a more reactive profile and changes in the deposition of ECM occur (Fig. 2) [1]. Collagen is intricately involved in this process, which includes overproduction, degradation, and altered composition detectable in pathophysiological samples [3]. Changes in the structure and/or deposition of collagen impact cellular signaling and tissue biomechanics which in turn can alter cellular responses in tissue, driving disease and inflammatory states [96]. Collagen contains multiple binding sites that serve as ligands for both cell surface receptors and signaling pathways involved in disease and aging-related inflammation. MMP-induced disruptions of these binding sites lead to chronic inflammation [108, 109]. Thus, even slight changes in ECM early on in disease progression may be overlooked as drivers of neurodegeneration.

CNS neurodegenerations often coincide with vascular pathologies such as compromised blood flow, leakage, and degradation of the blood-brain barrier [1]. Microglia, along with endothelial cells and astrocytes are key cellular contributors to the stability of the blood-brain barrier in the CNS, and changes in activity of these cell types during disease can alter ECM within the neurovascular unit, in particular collagen IV of the basement membrane, and disrupt barrier function [46–49]. In systemic vascular diseases such as atherosclerosis, damage to ECM in vascular walls contributes to the progression of disease (Fig. 2) [110]. Atherosclerosis is also associated with increased vessel stiffness possibly due to large deposits of collagen [110]. Recently, a potential role for complement factors C3 and C4 and ECM in the vascular etiology of neurodegenerative diseases has emerged. The unexpected observation that C3 and C4 deposit in vessel walls and colocalize with collagen has unearthed a potential interaction between complement proteins and collagen both in disease and in aging [111], and a similar process may lead to accumulation of complement factors in collagen-rich Bruch’s membrane in the retina during age-related macular degeneration. In CNS conditions such as Alzheimer’s Disease, early microglial activation is thought to be an early driver of neurodegeneration [112, 113]. Activated microglia promote phagocytosis of neurons and also contribute to the breakdown of the blood-brain barrier [113]. One mechanism for microglial activation depends on the binding of C3 to C3 receptors present on the microglial cell surface [114]. Thus, damaged collagen present in blood vessels due to aging or disease may act as a novel C3 reservoir, which may trigger early microglial activation and drive neurodegeneration.

Microglial cells also influence ECM composition through their expression and release of cytokines and MMPs [56]. Microglia are highly mobile cells that migrate during CNS development and in injury. Migration of microglia is in part governed by the surrounding ECM milieu, including via cell binding to integrin receptors on collagen in the ECM [115]. In CNS disease or injury, microglia become reactive and increase expression of MMPs [54, 116], changing the composition of the local ECM with downstream effects on other cell types, including the migration of oligodendrocytes and astrocyte function [34, 117].

The CNS lacks the intrinsic capability to regenerate, in part due to microenvironmental factors that increase local inflammation and reactive gliosis [118]. Pathological changes in ECM collagen may contribute to the limited capacity for regeneration in the CNS through perturbed signaling. A major component of astrocyte-mediated ECM deposition is collagen, which may promote ECM deposition and impact regeneration in the CNS [119]. As matrix collagen degrades, the formation of a glial, fibrous complex by reactive astrocytes secreting an overabundance of collagen type IV creates a barrier to axon repair and regeneration in the CNS by concentrating inhibitory molecules like proteoglycans and semaphorins and inducing migration of inflammatory microglia and other immune cells [120–123]. Thus, this hypertrophic glial complex (typically called a glial scar) provides not only a biomechanical barrier to regeneration but a biochemical one as well [45]. In optic nerve degeneration, changes in collagen alignment and stiffness in the eye’s sclera (which shapes the eye) and in the head of the optic nerve through which axons pass on the way to the brain impact progression through increased inflammation [124].

In contrast to the CNS, the peripheral nervous system has a far greater capacity for neuronal repair. Molecular signaling pathways such as integrins play an important role in the spontaneous regeneration of peripheral axons [125]. Collagen is also highly upregulated after peripheral nerve injury and synthesized by Schwann cells and fibroblasts [126, 127]. The high levels of collagen at the site of peripheral nerve injury could facilitate important axonal integrin signaling required for regeneration and may be indicative of a more important role of collagen than previously appreciated. Regeneration in non-mammalian species such as the zebrafish is strongly ECM-dependent; conditions that favor regeneration are rich in numerous collagens, including collagen XII, suggesting a pro-regenerative capacity for certain collagen sub-types [128, 129]. Current regenerative strategies have focused on modulating growth factor signaling, regeneration-associated genes, glial-mediated axon regeneration, cell replacement, and peripheral nervous tissue grafting [1], all with limited success. None leverage the complex signaling capabilities of the ECM and in particular, collagen.

Throughout the body, including in the CNS, the biomechanical properties of tissue can impact cellular signaling and cell recruitment to the site of damage or disease. After an injury in the CNS, the biomechanical stiffness of the tissue in the brain and spinal cord decreases, correlated with increased levels of ECM components including collagen IV and laminin [45]. If collagen becomes damaged enough to alter the stiffness of the local tissue area, a wave of mechanically induced signaling involving other cells could ensue. For example, traumatic injury to the brain involves a transient period of rapid neovascularization, increased vessel permeability, and accumulation of pro-angiogenic factors likely released by microglia cells [130]. These same cells likely contribute to tissue remodeling following injury through the secretion of proteases. The vascular basement membrane in the brain contains the heparan sulfate proteoglycan collagen XVIII, among other collagens, which contains a 20-kDa anti-angiogenic endostatin fragment (reviewed in [43]). Subsequent to the injury, parenchymal accumulations of collagen XVIII/endostatin accompany increased numbers of microglia expressing the collagen XVIII/endostatin [44]. This collagen XVIII-dependent process could contribute to counteracting the early angiogenic injury response to limit secondary injury.

### Repairing collagen in the ECM as a CNS therapeutic

For CNS regeneration, tissue biomechanics can impact the capacity for neuronal axon growth and guidance. Thus, maintaining intrinsic ECM biomechanical properties may be a potential therapeutic avenue. Just as damage to the ECM is prohibitive to regeneration, therapies that rebuild the matrix hold great therapeutic potential to promote neural repair and regeneration across conditions including traumatic brain injury [23, 131]. This is especially so for therapies utilizing or mimicking types I and IV collagen, which are known for very low antigenicity and robust bioavailability [132, 133]. Since the collagen interactome and tissue reactivity are heavily governed by collagen structure in the ECM, a more prominent role of collagen in tissue ECM homeostasis is emerging. These observations open a potential therapeutic avenue for matrix repair early in CNS disease, well beyond the normal slow process that governs collagen turnover. Indeed, a promising area is the potential uses of ECM and ECM-derived peptides that improve neuronal regeneration and functional recovery [134]. Similarly, the implantation of collagen-rich scaffolds following brain surgery reduces microglial activation and inflammatory-related cytokines [135].

The signature and universal characteristic of collagen is its triple helical structure -- a set of three polypeptide chains comprising repeating sequences of glycine-x-y triplets where x and y often (but not always) represent proline and hydroxyproline [136]. Recent work from our laboratory and others supports the idea that rebuilding triplets damaged by protease activity can repair CNS tissue and promote neuronal survival in a broad spectrum of neurodegenerative conditions. For example, following optic nerve crush, injection of a collagen mimetic peptide (CMP) that intercalates into and reforms compromised triple helices promoted axonal outreach beyond the crush site and extended the length of intact axon segments [137]. Similarly, following the induction of optic nerve degeneration by elevated ocular pressure, CMP treatment restored functional axon transport to central brain targets [137, 138]. This is a critical finding, since degradation of axonal transport presages outright axon degeneration [139, 140]. CMPs have a protective and reparative influence on peripheral nerve damage as well, demonstrating trophic capacity for dorsal root ganglia (DRG) challenged by MMP-induced degradation of ECM and for the corneal nerve bed damaged by surface desiccation [1, 138]. Gels enriched with collagen type I similarly promote neurite extension from DRG explants [141]. The reparative influence of collagen segments is not limited to neurons. Segmented sequences of collagens IV, XV, and XVIII promote the growth of blood vessels and tumor cells and influence a variety of other cellular activities [142]. Synthesized CMPs target areas of collagen disruption associated with skin wounds by reforming the native triple helix through intercalating into disrupted collagen [143, 144].

There are multiple mechanisms through which repairing damaged triple helices in collagen could affect a therapeutic influence, especially since there are no natural mechanisms to do so. Due to its intrinsic structural properties, collagen confers significant amounts of stiffness to tissue, in concert with other constituents of the ECM [145]. Interestingly, a stiffer matrix reduces the potential for astrocyte reactivity and gliosis, which presage the formation of the inflammatory complex at CNS injury sites that is so inhibitive of regeneration [146]. Along these lines, unlike other tissues that scar, the brain and spinal cord demonstrate *diminished* elastic stiffness after acute injury, which coincides with increased astrocyte reactivity and inflammation even distal to the injury site [45]. In this way, repaired collagen leading to a stiffer matrix could help reduce the inflammatory response at the level of the macroenvironment in the neuronal milieu. At the microenvironment level, degradation of collagen by MMP activity can disrupt binding sites that otherwise inhibit local inflammatory signaling complexes. For example, collagen types I and III (and possibly IV) contain high-affinity binding sites for LAIR-1 (leukocyte-associated immunoglobulin-like receptor), which is expressed by most hematopoietic cells to attenuate their activation [147]. Intact triple helices in collagen crosslink LAIR-1 to inhibit immune cell activation, while reduced LAIR-1 binding sites in disrupted collagen has the opposing effect [108]. Interestingly, the sequence for the binding site for LAIR-1 (also called CD305) is conserved between collagen and the complement component C1q, the initiator of the classical complement pathway at the root of innate and adaptive immunity [148]. C1q mediates synapse elimination from dendritic arbors in neurons and is activated early in diseases such as Alzheimer’s [149]. Collagen and C1q partner to locally regulate LAIR-1 to avoid immune dysfunction. Similarly, intact collagen type I inhibits the secretion of interleukin-8 from neutrophils through interaction with integrin receptors [150]. The threshold for a local inflammatory response is breached with shortening of collagen strands through degradation in disease or injury [109].

## Conclusions

In summary, CNS ECM represents more than a passive, inert scaffold to support tissue; ECM is a diverse, dynamic, and highly bioactive substrate with roles in cellular biomechanics and differentiation as well as intercellular signaling. Although ECM collagen has distinct roles in tissues outside of the CNS, its integral role in the CNS during homeostasis and disease presents an opportunity. Here we highlight multiple actions of collagen, both at the macroscale (in tissue support and biomechanics) and at the microscale (ligand binding capacity) in the CNS. The structure of collagen is important in rendering its highly functional nature. Damage to CNS collagen is evident in disease and with aging and has major implications for driving neurodegeneration through its impact on inflammatory pathways in particular. The repair of collagen damage early might in fact reduce the number of patients who progress to a chronic disease state. Repairing damaged collagen in the CNS early using mimetic peptides represents an exciting new therapeutic avenue for treating and slowing neurodegenerative diseases, where the restoration of collagen structure at the microscale level could help to repair, protect, and even regenerate CNS neurons.

## Data Availability

Not applicable.

## Abbreviations

Aβ: Amyloid-beta
AD: Alzheimer’s Disease
APP: Amyloid precursor protein
CNS: Central nervous system
CSF: Cerebrospinal fluid
DDRs: Discoidin domain receptors
DRG: Dorsal root ganglia
ECM: Extracellular matrix
GAGs: Glycosaminoglycans
HSPs: Heat-shock proteins
HSPGs: Heparan sulfate proteoglycans
LAIR-1: Leukocyte-associated immunoglobulin-like receptor
MMP: Matrix metalloproteinase
OPC: Oligodendrocte precursor cells
PNN: Perineuronal net
PNS: Peripheral nervous system
RGD: Arginine-glycine-aspartate
